# Challenges of face identification with varied mask coverage in the post COVID-19 era

**DOI:** 10.3389/fpsyg.2025.1486808

**Published:** 2025-02-27

**Authors:** Yi-Lang Chen, Shu-Yu Wang

**Affiliations:** Department of Industrial Engineering and Management, Ming Chi University of Technology, New Taipei, Taiwan

**Keywords:** post COVID-19 era, mask coverage, face identification, accuracy, target sex

## Abstract

**Introduction:**

Recent studies have shown that wearing masks can influence face recognition abilities. During the COVID-19 pandemic, people became increasingly familiar with seeing masked faces, leading to a reduced familiarity with fully uncovered faces. With Taiwan now transitioning to a post-COVID-19 phase and the removal of mask mandates, this study investigates how varying levels of mask coverage affect face identification accuracy and response times.

**Methods:**

We examined three levels of mask coverage—full coverage (FC), coverage up to the middle of the nose bridge (MB), and coverage up to the bottom of the nose bridge (BB)—to determine their effects on identification performance. A computer-based simulation was conducted with 100 university students (50 men and 50 women), where participants completed 30 trials (5 trials for each mask coverage level across two target sexes). Each trial presented a masked target face corresponding to one of the three coverage levels, alongside four full-face images. Participants were instructed to choose the image that best matched the masked target face, with an option to select “None” if no match was found.

**Results:**

The findings indicate that faces with FC were identified both faster and more accurately, while those with MB coverage were the most challenging and time-consuming to recognize, particularly for female targets. The performance with BB coverage was intermediate between the other two levels.

**Conclusion:**

This study highlights a notable shift in face identification processes in the aftermath of the pandemic, with FC now leading to quicker and more accurate recognitions, suggesting a significant adaptability in human perceptual mechanisms. These results emphasize the importance of further research into face recognition as we continue to adapt to the pandemic’s lasting effects on social interactions and identity verification.

## Introduction

Since the COVID-19 pandemic began in 2020, wearing masks has been one of the simplest and most effective measures to prevent coronavirus transmission. The United States Centers for Disease Control and Prevention (CDC, 2020) recommended that everyone cover their lower face in public settings. As the pandemic has subsided, mask mandates in many countries have been lifted. For instance, Taiwan ended its mask mandate in April 2023 and further relaxed the requirement in medical institutions by May 2024. This shift has introduced a new interpersonal challenge: recognizing faces as people transition from wearing masks to not wearing them.

While masks were effective in curbing the spread of COVID-19 during the pandemic, their widespread use significantly impacted daily life. Masks impaired face recognition, often causing individuals to misidentify strangers as familiar faces, which in turn fostered false memories (Iidaka et al., 2012). Additionally, mask-wearing, which conceals facial expressions, has been shown to influence emotional responses and social perceptions (Cartaud et al., 2020). For instance, Cannito et al. (2022) found that masks impaired the ability to assess a proposer’s (un)trustworthiness, thereby weakening its influence on decision-making in both intertemporal and risky choices. Similarly, Marini et al. (2021) reported that face masks significantly reduced people’s ability to distinguish between genuine and posed smiles, affecting the perceived authenticity of emotional expressions and potentially undermining social trust and rapport. Moreover, Stajduhar et al. (2022) observed that masks diminished the ability to accurately recognize emotional intensity, with children aged 6–14 experiencing a particularly sharp decline in facial identification skills. Additionally, mask-wearing complicated the recognition of emotions, gender, age, and identity (Fitousi et al., 2021). Public mask usage also presented challenges in situations requiring facial recognition, such as identity verification at border crossings or when purchasing age-restricted items like alcohol (Carragher and Hancock, 2020). Furthermore, masks were used by individuals to obscure their appearance while committing crimes (Southall and Van Syckle, 2020). In response to these challenges, an online system was developed to incorporate facial mask detection into attendance tracking based on facial recognition (Kamil et al., 2023).

Although facial recognition technologies, driven by advanced algorithms, are widely used for security and identity verification in public settings (Teboulbi et al., 2021; Kamil et al., 2023; Al-Dmour et al., 2023; Naseri et al., 2023), face identification in daily interpersonal interactions primarily relies on human capabilities rather than machines. Consequently, it is essential to understand the human factors involved in facial recognition. Face masks, which obscure the lower part of the face (mouth and nose), disrupt social interactions and hinder identification processes. Studies have investigated the impact of face masks on face-matching performance (Carragher and Hancock, 2020; Noyes et al., 2021). While individual differences in performance exist (Estudillo et al., 2021), masks generally impair overall face-matching accuracy (Carragher and Hancock, 2020; Noyes et al., 2021).

Face identification accuracy is strongly influenced by the familiarity of the face being recognized. Identifying unfamiliar faces is notably error-prone, even under ideal conditions (Burton et al., 2010; Kramer et al., 2018), and accuracy further diminishes in suboptimal scenarios (Fysh and Bindemann, 2017). For instance, approximately 20% of comparisons between two photographs taken just minutes apart result in errors (Burton et al., 2010). These difficulties are evident in tasks requiring comparisons of two images (Megreya and Burton, 2006; Burton et al., 2010) or matching an image to a live person (White et al., 2014). In contrast, familiarity significantly enhances recognition accuracy, likely due to differences in processing strategies. Ellis et al. (1979) previously demonstrated that internal facial features, such as the eyes and nose, are crucial for identifying familiar faces. For unfamiliar faces, however, recognition accuracy was comparable regardless of whether internal or external features were used. Interestingly, Carragher and Hancock (2020) reported that wearing masks substantially impairs face-matching performance, with similar levels of impairment observed for both familiar and unfamiliar faces. These findings suggest that familiarity not only improves face identification but also shifts the emphasis placed on specific facial features during recognition.

The distinction between upper- and lower-face features is particularly relevant in face identification under conditions of partial occlusion. Studies indicate that upper-face features, including the eyes and hairline, are more diagnostically valuable in recognition tasks. For example, Reynolds and Pezdek (1992) found that upper-face features had lower false alarm rates compared to lower-face features. Similarly, McDonnell et al. (2014) observed that participants fixated more quickly and for longer durations on upper facial features, such as the eyes and brows, particularly when recognizing same-race faces, which improved recognition accuracy. In the context of emotional facial expression identification, Bandyopadhyay et al. (2023) showed that accuracy peaked when only the eyes and mouth were visible, suggesting that other facial regions might serve as distractors. Masks, by occluding the lower half of the face—including the nose and mouth—further highlight the importance of upper facial features, as individuals must rely on visible regions for recognition (Noyes et al., 2021; Chen et al., 2023). These observations underscore the critical role of key features, especially the eyes, in both identity and emotion recognition. However, partial occlusions, such as masks ending at the nose bridge, can introduce ambiguity by exposing some lower-face features while disrupting holistic facial processing (Fitousi et al., 2021). These findings underscore the importance of investigating how different occlusion patterns affect the relative contribution of upper and lower facial regions in face identification.

Wearing a mask inevitably impairs face recognition, thereby affecting daily interpersonal communication (Marler and Ditton, 2021; Grahlow et al., 2022; Kalsotra et al., 2024). However, the process of face identification during the pandemic differs from that in the post-pandemic period. Early in the pandemic, people had to adjust from recognizing unobstructed faces to recognizing those partially covered by masks. This obstruction significantly impacted facial recognition. Chen et al. found that identification accuracy for faces with full mask coverage was significantly lower than for those with masks covering only up to the middle (MB) or bottom (BB) of the nose bridge. Interestingly, there was no difference in accuracy between the MB and BB levels, suggesting that reducing mask coverage to the BB level does not improve face identification.

**Figure 1:**
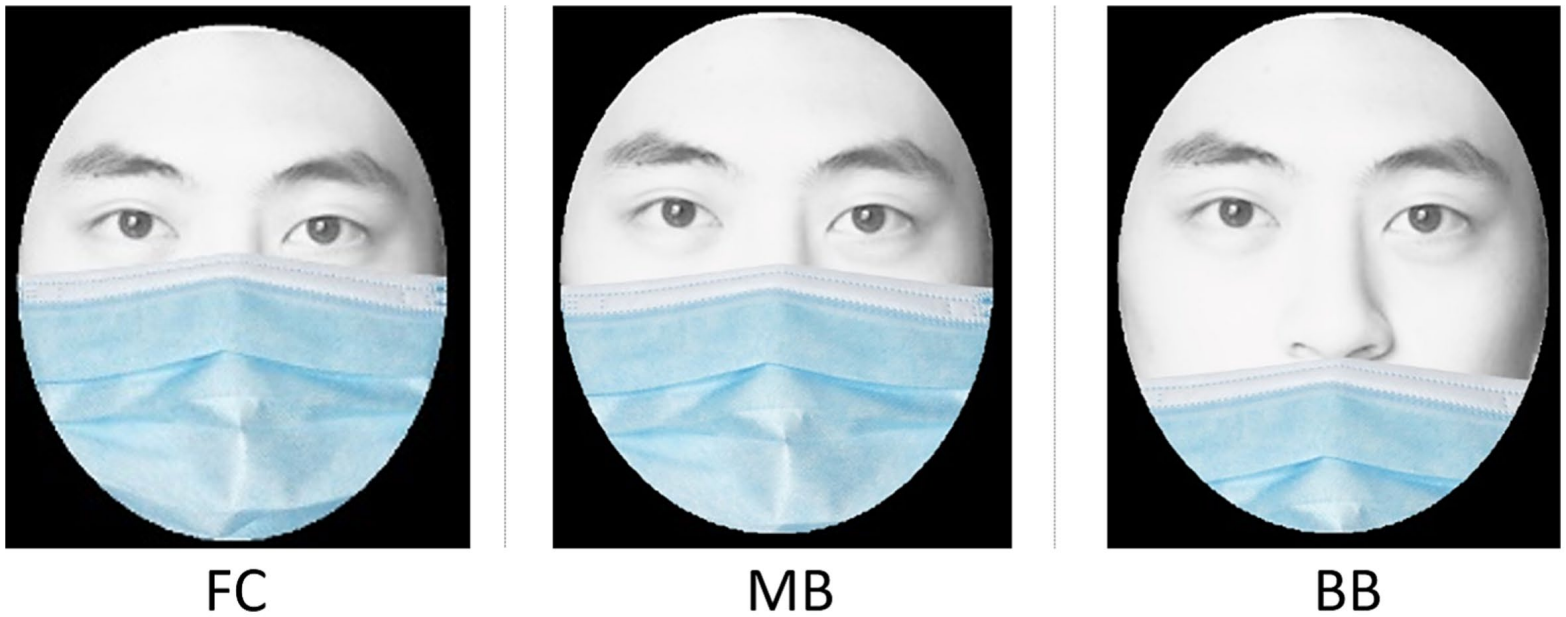
Three common mask coverage levels (FC, full coverage; MB, coverage up to the middle nose bridge; and BB, coverage up to the bottom nose bridge).

In the post-pandemic period, as people gradually remove their masks, the face identification process reverses, requiring recognition of uncovered faces based on the memory of familiar masked faces. Wearing masks impairs several functions, including speech, breathing, and overall comfort (Kumar and Lee, 2020; Rahne et al., 2021; Zhang et al., 2022), which can collectively impact daily communication. Although the CDC provided guidelines for proper mask-wearing, it was observed that individuals often modified mask coverage (Figure 1) to enhance comfort or facilitate easier identification by others. For instance, a survey in Indonesia revealed that only 34.3% of respondents wore masks correctly (Siahaan et al., 2021), while Ganczak et al. (2021) found that approximately 50% of improper mask usage involved uncovered noses.

The COVID-19 pandemic spurred numerous studies on face recognition with masks, but few have explored the reverse scenario: how different levels of mask coverage affect face identification once masks are removed. To address this gap, we recruited 100 participants (50 men and 50 women) to assess face identification accuracy and response time across three mask coverage levels (FC: full coverage; MB: coverage up to the middle of the nose bridge; and BB: coverage up to the bottom of the nose bridge; Figure 1). We hypothesized that as mask coverage decreases, both face identification accuracy and identification time would improve.

## Materials and methods

### Participants

We recruited 100 university students (50 men and 50 women) with a mean age of 21.2 years (SD = 2.3) for men and 22.0 years (SD = 2.8) for women. *A priori* power analysis was conducted using G*Power (Version 3.1.9.7) to determine the required sample size for a repeated measures analysis of variance (ANOVA) with a within-between interaction. With a desired power of (1 − *β*) = 0.8, an *α*-error probability of 0.05, a medium effect size (*f* = 0.35), two groups (male and female), and six measurements (two target sexes and three masking levels), the power analysis determined a minimum required sample size of 66 participants. Our actual sample of 100 participants exceeded this requirement, ensuring sufficient statistical power for the study. Participants were recruited via online advertisements posted on Google Forms. The advertisement included a brief study description, eligibility criteria, and compensation details. Each participant received a monetary compensation of US$6 for completing the study, which took approximately 20 min in total. All participants were familiar with computers, a requirement for the test. None of the participants had visual impairments, such as color blindness or color weakness. Only individuals with normal vision, either unaided or corrected, were included in the study. Informed consent was obtained from all participants prior to their involvement, and the study received approval from the Ethics Committee of Chang Gung Memorial Hospital, Taiwan.

### Stimuli

**Figure 2:**
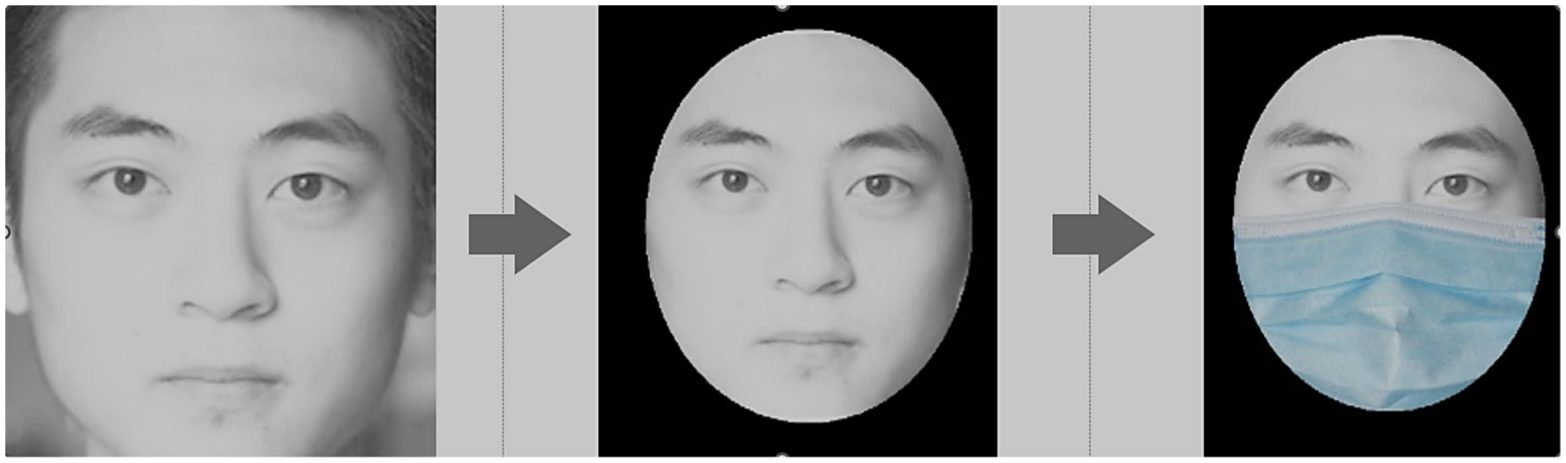
Face image conducted by the test.

To investigate the effect of mask coverage on face identification, we utilized three coverage levels (FC, MB, and BB) as outlined in Chen et al. (2023). The face images used in the test were sourced from the Asian Face Age Dataset (from CVPR 2016 open access, Niu et al., 2016). We randomly selected 40 frontal images of male and female faces (20 each), aged 18–24 years, from which 10 images (five men and five women) were chosen as target images. These images were edited using Adobe Illustrator and Adobe Photoshop 23.5 (Adobe Systems, San Jose, CA, USA), converted to greyscale, and standardized in brightness and contrast to eliminate visual interference. Only facial features were retained in the images, with all non-facial elements removed (Figure 2). Masks were then superimposed on the faces to create the three coverage levels. In our study, we exclusively focused on internal features when examining unfamiliar faces to align with previous research and minimize the influence of familiarity, as internal features are less impacted by familiarity effects (Ellis et al., 1979; Carragher and Hancock, 2020; Noyes et al., 2021).

A total of 30 questions were developed, each presenting five male and five female faces across the three coverage levels. Participants were asked to identify the correct face from four candidate images. In addition to the 10 target images, 30 images were included as distractors.

### Experimental design and procedure

For each mask coverage level, 10 face images (five men and five women) were used, resulting in 30 questions across the three levels. This generated a total of 3,000 data points (100 participants × 3 coverage levels × 10 questions), capturing both the participants’ responses and their identification times. The order of testing was fully randomized to prevent learning effects or cumulative bias. The experimental task was conducted using a custom-developed web-based application built with HTML5, JavaScript, and CSS. The program was presented on a wall-mounted 55-inch LCD display (1,920 × 1,080 pixels resolution, 60 Hz refresh rate). Participants provided responses using a standard QWERTY keyboard and an optical mouse placed on a designated response station. The test room was maintained under standardized lighting conditions (approximately 500 lux) to ensure consistent visibility of the displayed images across all sessions.

**Figure 3:**
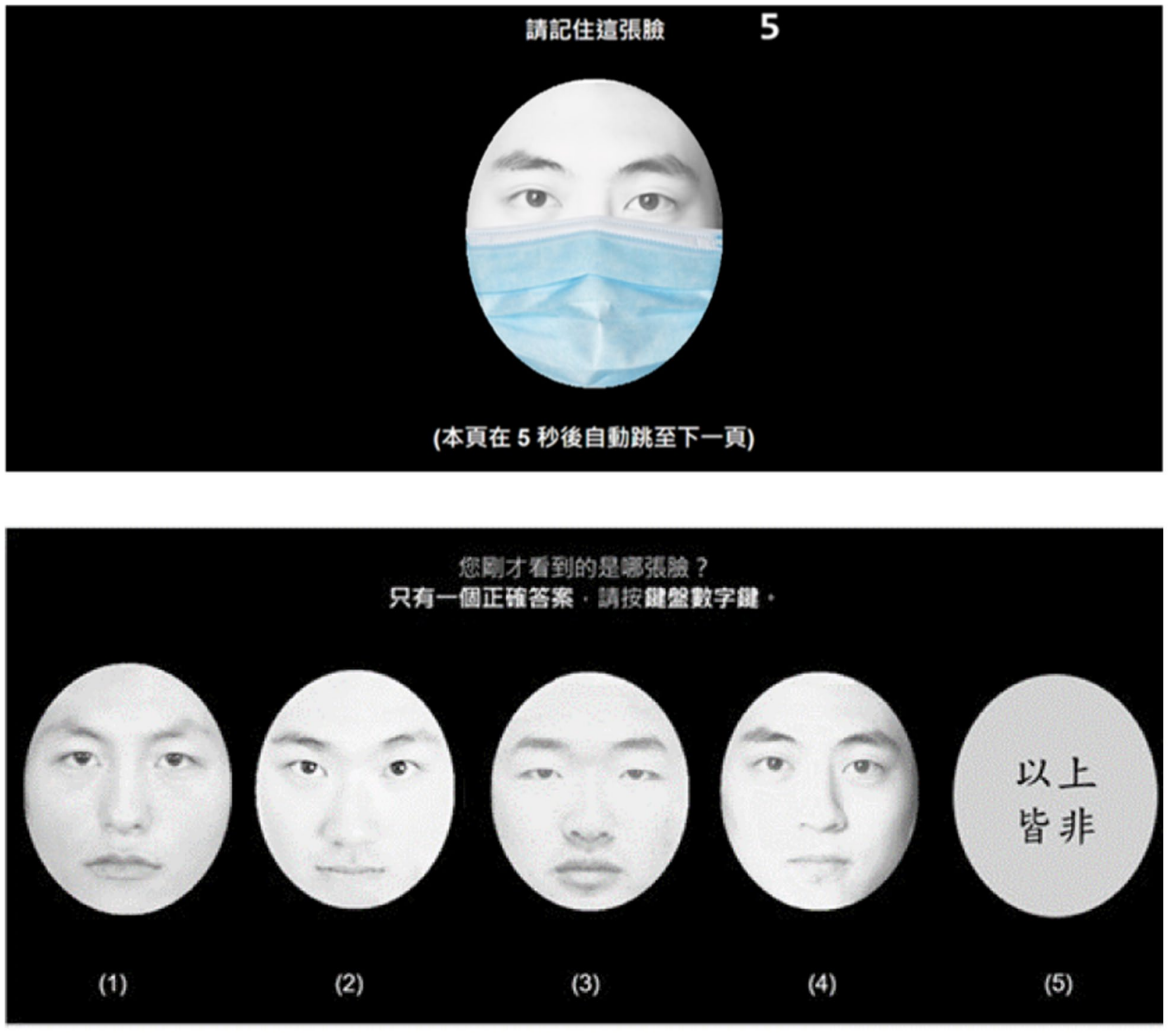
A pilot test format similar to the final test with a full coverage level.

The test was conducted in a quiet, isolated room. Participants stood and given instructions displayed on the screen before starting the test. They were allowed to adjust their feet and keyboard positions to ensure that their line of sight was perpendicular to the screen and they were comfortable throughout the experiment. A two-stage practice session preceded the data collection. The first stage familiarized participants with the use of the computer keyboard and mouse, while the second stage involved a pilot test with questions similar in format to the final test. After the practice session, an alert box notified participants that the formal test was about to begin. Upon pressing the OK button, an 8-s countdown appeared on the screen, providing time for participants to answer each question. If a participant failed to respond within the allotted time, the answer was marked as invalid, and the test automatically proceeded to the next question. Participants were required to select the correct face from four options for each of the 30 questions. If none of the options matched the target face, participants could choose “None,” as shown in Figure 3.

### Statistical analysis

Statistical analyses were conducted using SPSS version 22.0 (SPSS, Inc., Chicago, IL, USA), with the significance level set at *α* = 0.05. Normal distribution of numerical variables was assessed using the Kolmogorov–Smirnov test, while homogeneity of variances was evaluated using Levene’s test to ensure robustness of the analysis. Descriptive statistics, including means and standard deviations, were calculated to summarize the data. To evaluate the impact of participant sex, mask coverage level (FC, MB, and BB), and target sex on face identification accuracy and response time, a three-way ANOVA was performed. Each participant was treated as a block and exposed to all treatment combinations in a randomized sequence. Participant sex was analyzed as a between-subject factor, while mask coverage level and target sex were treated as within-subject factors. Tukey’s Honestly Significant Difference test was applied for *post-hoc* comparisons. To determine the practical importance of any significant independent variable, the power value was calculated based on Cohen’s guidelines (Cohen, 1988). An effect size of around 0.2 represents a small effect, around 0.5 indicates a medium effect, and around 0.8 reflects a large effect. To further explore the data, independent *t*-tests were performed for detailed comparisons of each testing combination, and Cohen’s *d* was calculated to quantify the effect sizes for significant differences.

## Results

### Three-way ANOVA

Out of the 3,000 responses collected, only 23 exceeded the 8-s time limit, resulting in a validity rate of 99.2%. The results of the three-way ANOVA for face identification accuracy and response time are presented in Tables 1, 2, respectively. Mask coverage levels (*p* < 0.001) and target sex (*p* = 0.003) had significant effects on face identification accuracy, while all independent variables influenced response time (participant sex and mask coverage level, *p* < 0.001; target sex, *p* = 0.034). Women had a shorter identification time (3.27 s) compared to men (3.78 s). According to Tukey test (Table 3), face identification accuracy was significantly lower at the MB level (78.1%) compared to the FC (85.4%) and BB levels (82.5%), with no significant difference between the non-MB levels. Identifying faces under the MB condition took longer (3.71 s) than under the FC condition (3.33 s). Additionally, significant interactions were observed between mask coverage level and target sex for both accuracy (*p* = 0.044) and response time (*p* = 0.007), suggesting that further cross-analysis is warranted.

**Table 1:** Three-way ANOVA results of face identification accuracy.

Sources	SS	df	MS	*F*	*p*-value	Power
Participant sex (PS)	0.013	1	0.013	0.441	0.507	0.102
Coverage level (CL)	0.540	2	0.270	9.109	<0.001	0.975
Target sex (TS)	0.256	1	0.256	8.639	0.003	0.835
PS × CL	0.008	2	0.004	0.137	0.872	0.071
PS × TS	0.010	1	0.010	0.324	0.570	0.088
CL × TS	0.186	2	0.093	3.131	0.044	0.601
PS × CL × TS	0.025	2	0.013	0.425	0.654	0.119

**Table 2:** Three-way ANOVA results of face identification time.

Sources	SS	df	MS	*F*	*p*-value	Power
Participant sex (PS)	38.423	1	38.423	38.226	<0.001	1.000
Coverage level (CL)	14.037	2	7.019	6.983	0.001	0.926
Target sex (TS)	4.547	1	4.547	4.524	0.034	0.615
PS × CL	0.001	2	<0.001	<0.001	0.999	0.050
PS × TS	0.163	1	0.163	0.163	0.687	0.069
CL × TS	9.971	2	4.986	4.960	0.007	0.810
PS × CL × TS	0.494	2	0.247	0.246	0.782	0.089

**Table 3:** Tukey test results of identification accuracy and response time.

Mask coverage levels	Accuracy (%)	Tukey test	Time (s)	Tukey test
Full coverage (FC)	85.4 (17.9)	A	3.33 (0.95)	A
Middle of nose bridge (MB)	78.1 (18.4)	B	3.71 (1.09)	B
Bottom of nose bridge (BB)	82.5 (15.5)	A	3.54 (1.07)	AB

Data (mean with standard deviation in parentheses) with the same letter do not differ in Tukey test.

### Cross analysis

**Figure 4:**
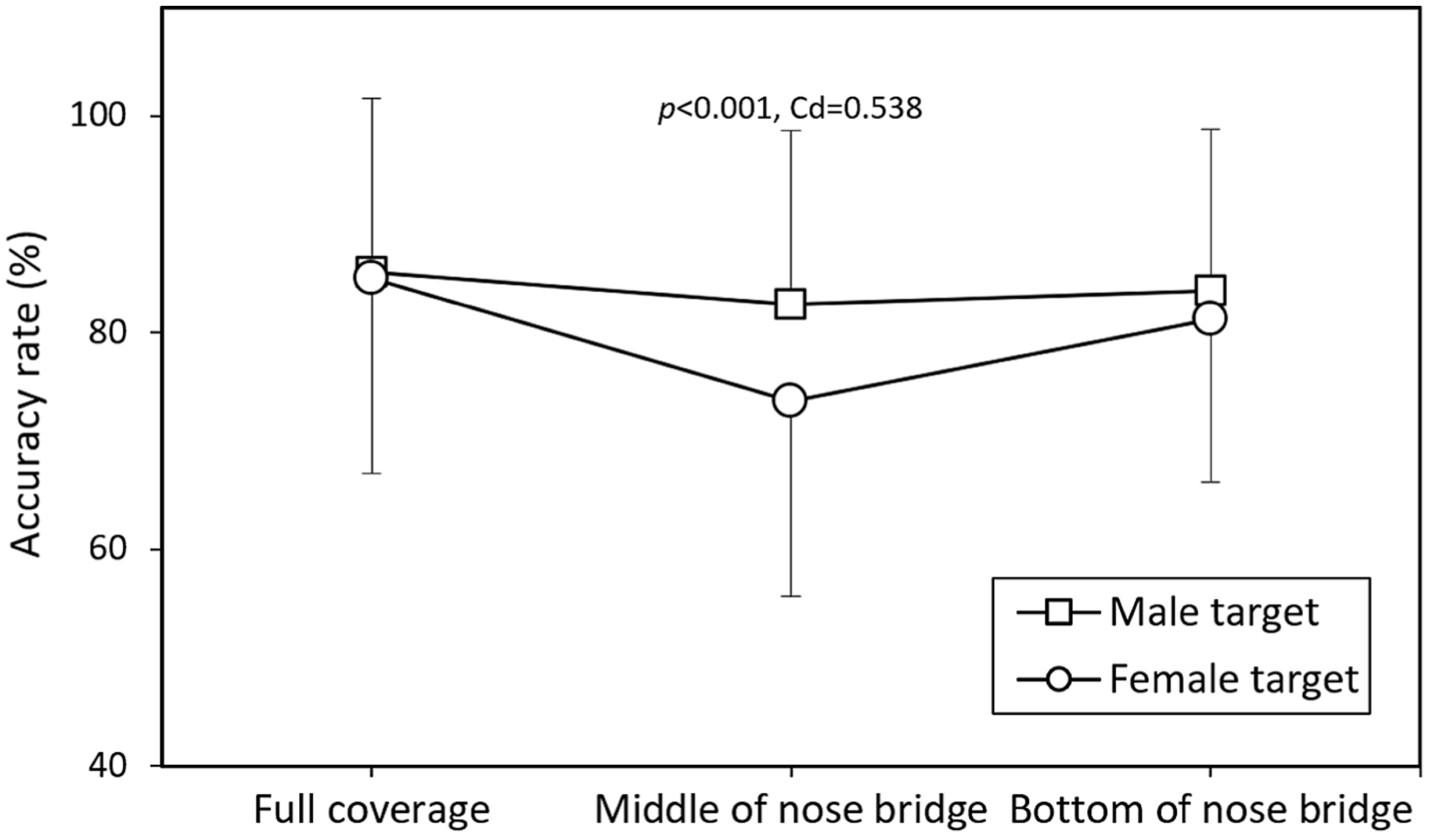
Interaction effects of mask coverage level and target sex on identification accuracy rate (Data presented as means with standard deviations), with Cohen’s *d* (Cd) reported for significant differences.

**Figure 5:**
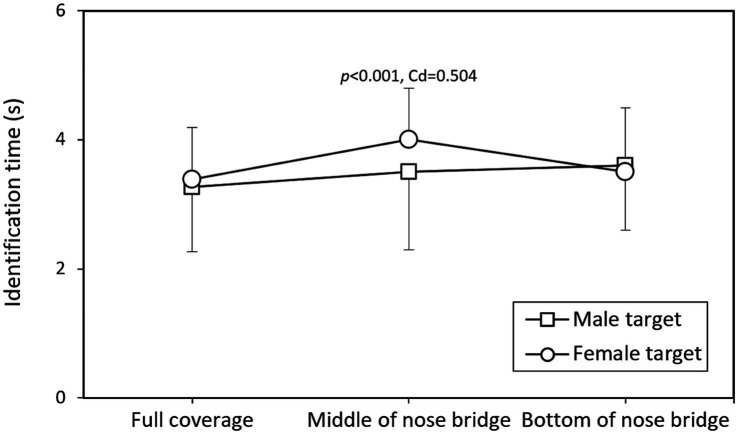
Interaction effects of mask coverage level and target sex on the identification time (Data presented as means with standard deviations), with Cohen’s *d* (Cd) reported for significant differences.

**Figure 6:**
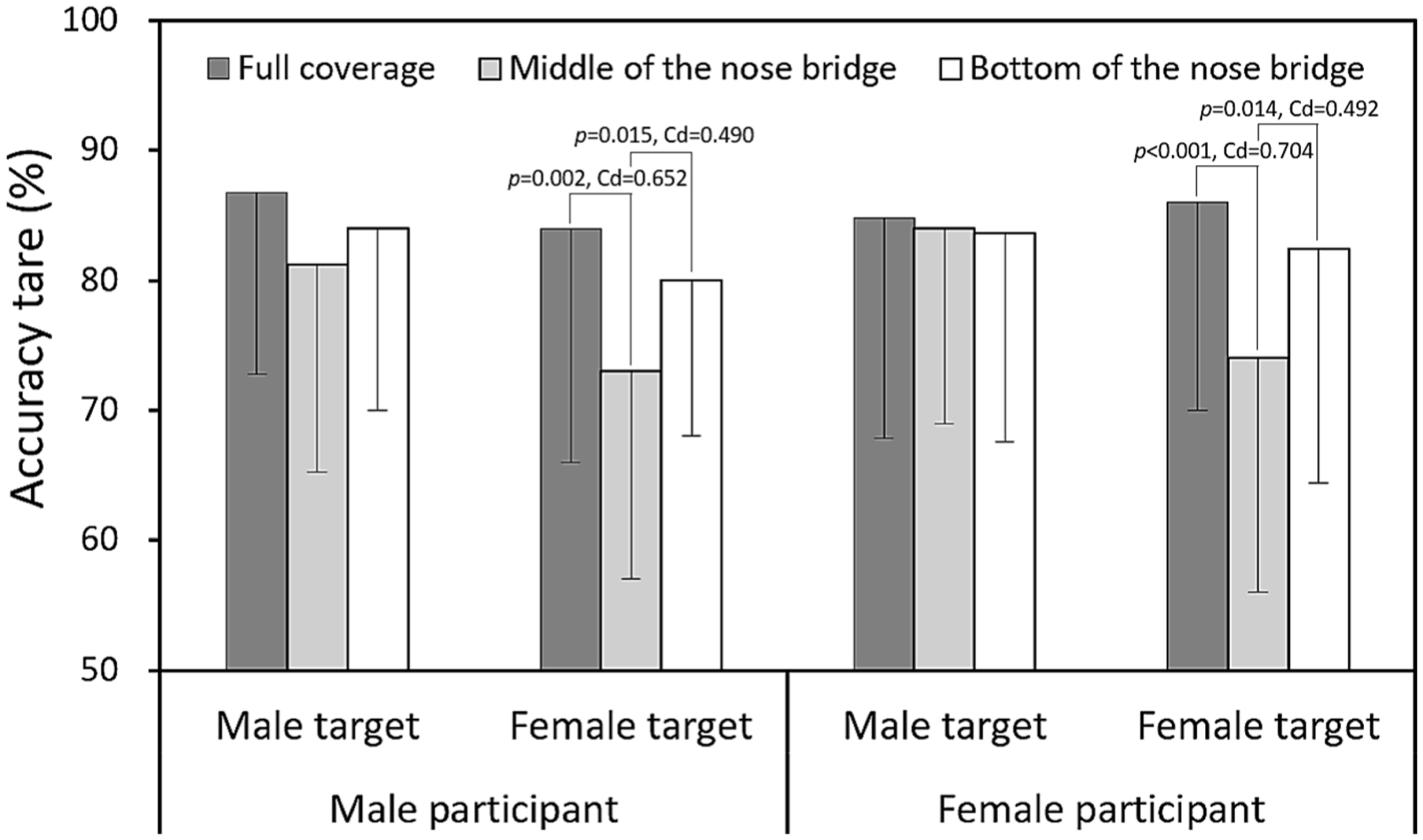
Accuracy rates across different mask coverage levels, compared by target gender and participant gender (Data presented as means with standard deviations, and Cohen’s *d* [Cd] reported for significant differences).

**Figure 7:**
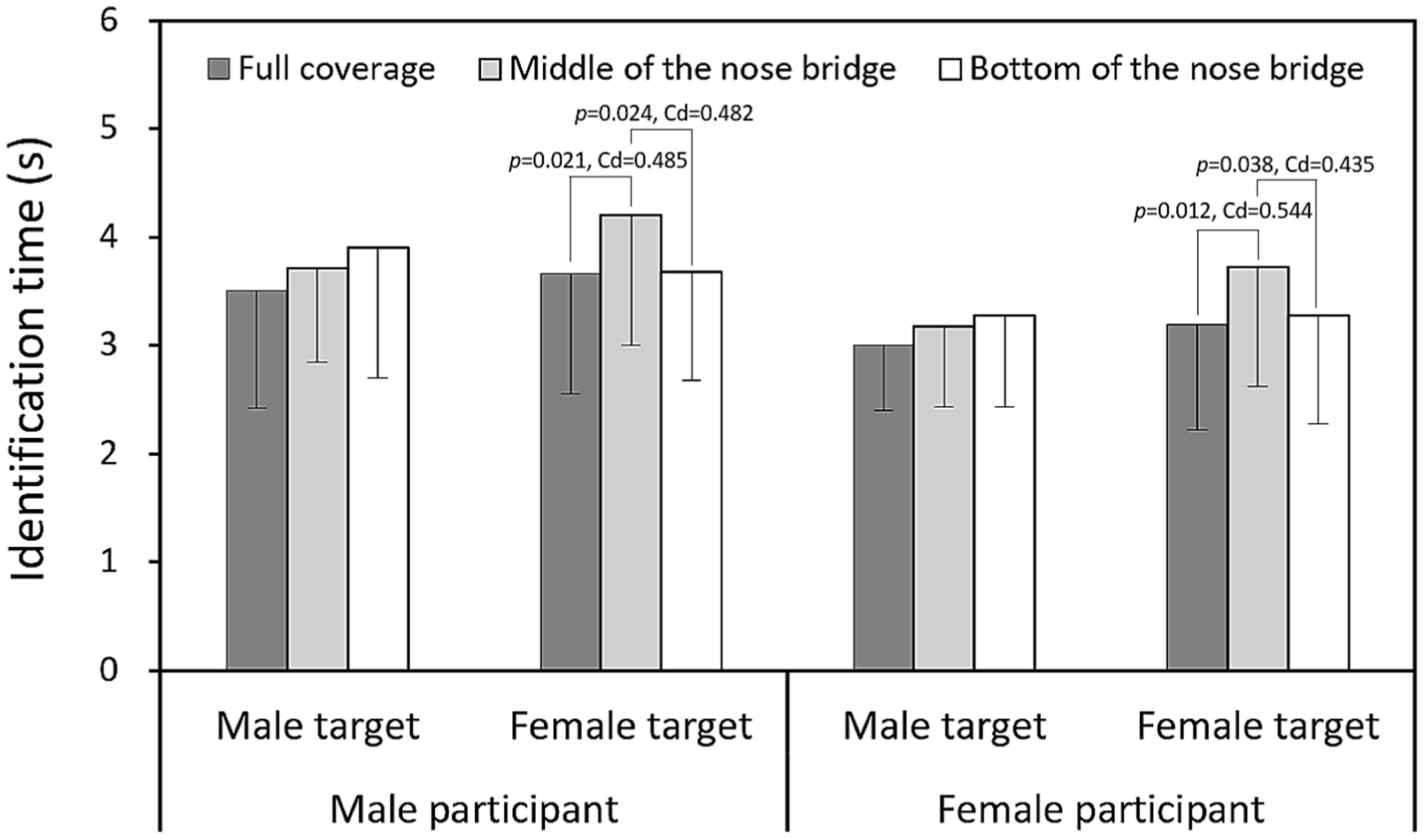
Identification times across different mask coverage levels, compared by target gender and participant gender (Data presented as means with standard deviations, and Cohen’s *d* [Cd] reported for significant differences).

Figures 4, 5 depict the interaction effects of mask coverage level and target sex on identification accuracy and response time, respectively. The data indicate that identifying female targets with masks at the MB level was significantly more difficult (*p* < 0.001, Cohen’s *d* = 0.538) and required more time (*p* < 0.001, Cohen’s *d* = 0.504) compared to male targets. When the comparative results in Table 3, which present data averaged across other variables (participant gender and target gender), are considered, Figures 6, 7 further illustrate accuracy rates and identification times across different levels of mask coverage, analyzed by both target gender and participant gender. Overall, both male and female participants demonstrated the lowest recognition accuracy and the longest identification times when identifying female faces with MB-level mask coverage. In contrast, no significant differences were observed in the identification of male faces across the three mask coverage levels.

## Discussion

This study examined the effects of different mask coverage levels on face identification accuracy and response time in the post-COVID-19 context. The findings demonstrate significant impacts of mask coverage and target sex on both accuracy and response time, shedding light on the emerging challenges of facial recognition as society adapts to new mask-wearing norms. Notably, faces with FC level were identified both the fastest and most accurately, while identifying female targets proved significantly more difficult and time-consuming than identifying male targets. These results diverge from our initial expectations and contrast with previous findings during the pandemic (Chen et al., 2023), where the extent of mask coverage was not directly linked to identification accuracy. This discrepancy suggests that participants’ familiarity with facial mask features and mask-wearing styles may play a more pivotal role than previously thought, warranting further investigation.

### Impact of mask coverage levels

Contrary to our hypothesis and previous findings, the current study revealed that FC masks led to the fastest and most accurate identifications—a stark contrast to our earlier work (Chen et al., 2023), where FC resulted in lower identification accuracy compared to partial coverage (MB and BB levels). This unexpected outcome suggests a significant shift in face processing strategies in the post-pandemic period. It is possible that when faced with FC masks, participants focused more intensely on the visible features, particularly the eyes. Prior research has consistently highlighted the critical role of the eyes in face identification, with numerous studies demonstrating that recognition of the upper face is more accurate than that of the lower facial regions (Davies et al., 1977; Dal Martello and Maloney, 2006). Specifically, the eyes have been identified as the most crucial feature for facial recognition (Roberts and Bruce, 1988; McDonnell et al., 2014), surpassing lower facial features such as the nose, mouth, and chin in importance.

Recent studies on mask-wearing during the COVID-19 pandemic have revealed notable perceptual adaptations. Facial coverings not only disrupt traditional recognition of identity and emotional expressions but may also enhance processing of the eye region. Villani et al. (2022) proposed that masks increase the prominence of the eyes, serving as a reliable cue for directing social and spatial attention. This interpretation is supported by findings from Stajduhar et al. (2022), who reported increased attentional focus on the eye region during mask use. Noyes et al. (2021) also found that occlusion of the lower face enhances the salience of the eyes for identity judgments. This effect may be particularly pronounced with FC masks. Furthermore, fully covered faces might provide less ambiguous visual input compared to partially covered faces. Fitousi et al. (2021) suggested that partial occlusion can introduce more errors in face processing tasks than full occlusion. Our findings align with this perspective, showing that mask coverage at the MB level, which partially exposes the nose, introduces additional distracting visual information. This interference leads to lower identification accuracy and longer identification times compared to other mask coverage levels.

The discrepancy between our current and previous findings underscores the dynamic nature of face recognition processes and their capacity to adapt to changing environmental demands. Freud et al. (2022) noted that prolonged exposure to masked faces during the COVID-19 pandemic led to a recalibration of face processing mechanisms. Our results further support this adaptive process and suggest that these changes may continue in the post-pandemic period. Interestingly, the MB level was the most challenging for identification in this study, indicating that partial facial occlusion may create more ambiguity in recognition than full coverage, likely due to the disruption of holistic face processing, leading to the adoption of new recognition strategies (Stajduhar et al., 2022).

### Participant sex effect

Our findings show that while participant sex did not significantly impact identification accuracy (Table 1), it did influence response time (Table 2). This contrasts with previous studies (Wong and Estudillo, 2022; Chen et al., 2023), which reported sex differences in either accuracy or response time. Several factors might explain this discrepancy. First, our study utilized a more diverse set of stimuli, potentially reducing gender-specific biases in face recognition (Johnson et al., 2020). Second, the design of our task emphasized rapid decision-making, which may have lessened the influence of sex-related processing differences on accuracy. Additionally, the observed sex-based difference in response time, without a corresponding difference in accuracy, aligns with recent research suggesting that sex effects in face recognition tasks may be more nuanced than previously understood (Huc et al., 2023). Furthermore, sociocultural factors and individual exposure to diverse faces may have a greater impact on face recognition abilities than biological sex (Scherf et al., 2017).

### Target sex effect

Our study found that identifying female targets was significantly more challenging and time-consuming than identifying male targets, particularly when their faces were partially masked at the MB level, as shown in Figures 6, 7. This result is intriguing but difficult to compare with previous research, as there has been limited direct examination of this specific topic. However, mask coverage could potentially obscure facial features that are more critical for recognizing female faces (Bruce et al., 1993). Existing research, including the eye-tracking study by Heisz et al. (2013), has shown that individuals adopt different scanning patterns when viewing faces of different genders. These variations in visual processing strategies are linked to the target faces themselves, reflecting inherent differences in facial feature configurations and perceptual characteristics between male and female faces (Little et al., 2011). While previous studies have primarily focused on gender-based differences in facial structure and processing, our study advances this understanding by investigating how these differences interact with mask coverage levels, ultimately affecting face identification accuracy and response times (Noyes et al., 2021; Stajduhar et al., 2022).

Research has also indicated that facial recognition algorithms struggle more with identifying women wearing masks compared to men, which may partially explain similar challenges in human recognition. Damer et al. (2020) showed that facial recognition algorithms generally perform worse on masked faces, highlighting the difficulties posed by facial occlusion. Similarly, Dhamecha et al. (2014) found that various forms of facial disguise, including partial covering, significantly impact both human and machine recognition capabilities. The complexity of facial recognition is further underscored by Ranjan et al. (2018), who considered factors such as sex in facial analysis tasks. Although Albiero et al. (2020) identified gender-based differences in facial recognition accuracy, their study did not specifically address the impact of masks. While existing literature suggests that the interaction between mask-wearing and gender in facial recognition is complex and influenced by multiple factors (Dhamecha et al., 2014; Masi et al., 2018; Ngan et al., 2020), it does not directly support the notion that women with masks are more difficult to identify than men with masks. This interaction between gender, mask-wearing, and facial recognition accuracy remains an area requiring further targeted research.

### Interaction between mask coverage and target sex

The significant interaction between mask coverage level and target sex on both accuracy and response time (Figures 4, 5) represents a novel finding that was not observed in our previous study (Chen et al., 2023). This interaction indicates that the effect of mask coverage on face identification varies by gender, introducing additional complexity to the face recognition process in the post-pandemic era. This observation is consistent with recent research by Huc et al. (2023), who reported that masks had a more pronounced impact on recognizing female facial expressions compared to male expressions. The interaction we identified may reflect differences in the salience of facial features between males and females when partially occluded by masks.

### Contributions and limitations

The most striking aspect of our findings is the apparent reversal in the effect of mask coverage on face identification accuracy, indicating a significant adaptation in face recognition processes during and after the pandemic. The improved performance with fully covered faces suggests that individuals may have developed new strategies for identifying masked faces, possibly by relying more on visible features such as the eyes. This adaptation aligns with the concept of perceptual learning, where repeated exposure to certain stimuli enhances recognition abilities (Goldstone, 1998). Our results imply that societal adaptation to mask-wearing may have altered face processing mechanisms, leading to better performance with fully masked faces. These findings have important implications for the development and implementation of face recognition technologies in the post-pandemic era. The improved accuracy with fully masked faces suggests that these systems may need recalibration to better match human performance under current conditions. Additionally, the challenges associated with partial mask coverage (MB level) and the observed gender differences in identification accuracy underscore the need for more nuanced approaches to face recognition in both technological and social contexts.

However, our study has several limitations. The sample was limited to university students, which may not be representative of the broader population. This age limitation is particularly important, as recent research by Di Crosta et al. (2023) has shown significant age-related differences in how younger and older adults perceive and evaluate masked faces. Older adults may process facial information differently and exhibit varying degrees of adaptability to new perceptual challenges. As a result, the face identification strategies observed in our younger sample may not be generalizable to older populations. Future research should incorporate a broader age range and more diverse demographics to better understand these potential age-related differences in strategy development and adaptation. Additionally, the study used a specific set of face images, so results may vary with different stimuli. The facial images presented were cropped to exclude the full head and hair. Since face perception is inherently holistic, the absence or partial concealment of hair due to masks may disrupt contextual processing. This potential limitation should be taken into account when interpreting the results. Longitudinal studies tracking changes in face identification abilities as mask-wearing norms continue to evolve would also be valuable. Furthermore, exploring the impact of different types of masks and varying levels of familiarity with target faces could provide further insights into the complex dynamics of face recognition in the post-pandemic context.

## Conclusion

This study offers valuable insights into the challenges of face identification in the post-COVID-19 era, uncovering significant effects of mask coverage levels and target sex on both accuracy and response time. Contrary to our expectations and prior findings, full face coverage led to the most accurate and fastest identifications, whereas partial coverage (up to the middle of the nose bridge) posed the greatest difficulty. Additionally, female faces were generally harder to identify than male faces. These findings underscore the dynamic nature of face recognition processes and their capacity to adapt to changing environmental conditions. The observed reversal in the effect of mask coverage on identification accuracy points to a significant adaptation in face recognition mechanisms during and after the pandemic. This adaptability has crucial implications for our understanding of face processing and how these mechanisms adjust in response to sustained changes in facial appearance norms.

## Data Availability

The original contributions presented in the study are included in the article/supplementary material, further inquiries can be directed to the corresponding author.
